# SENP5 mediates breast cancer invasion via a TGFβRI SUMOylation cascade

**DOI:** 10.18632/oncotarget.1783

**Published:** 2014-02-18

**Authors:** Rivki Cashman, Helit Cohen, Rotem Ben-Hamo, Alona Zilberberg, Sol Efroni

**Affiliations:** ^1^ Faculty of Life Sciences, Bar Ilan University, Ramat-Gan, Israel

**Keywords:** SENP5, Breast Cancer, SUMOylation, TGFBR1

## Abstract

Identifying novel mechanisms, which are at the core of breast cancer biology, is of critical importance. Such mechanisms may explain response to treatment, reveal novel targets or drive detection assays.

To uncover such novel mechanisms, we used survival analysis on gene expression datasets encompassing 1363 patients. By iterating over the compendia of genes, we screened for their significance as prognosis biomarkers and identified SUMO-specific protease 5 (SENP5) to significantly stratify patients into two survival groups across five unrelated tested datasets. According to these findings, low expression of SENP5 is associated with good prognosis among breast cancer patients.

Following these findings, we analyzed SENP5 silencing and show it is followed by inhibition of anchorage-independence growth, proliferation, migration and invasion in breast cancer cell lines. We further show that these changes are conducted via regulation of TGFβRI levels. These data relate to recent reports about the SUMOylation of TGFβRI. Following TGFβRI changes in expression, we show that one of its target genes, *MMP9*, which plays a key role in degrading the extracellular matrix and contributes to TGFβ-induced invasion, is dramatically down regulated upon SENP5 silencing.

This is the first report represents SENP5-TGFβ-MMP9 cascade and its mechanistic involvement in breast cancer.

## INTRODUCTION

Breast Cancer is the most frequent malignant neoplasm among women in the western world, with approximately 234,580 new cases in 2013 and 40,030 estimated deaths in the United States alone [1]. In these patients, it is not the primary tumor, but its metastases at distant sites that are the main cause of death. As it is not possible to accurately predict the risk of metastasis development in individual patients, many women who would be treated by local treatment alone, which includes surgery and radiotherapy, will needlessly be ‘over-treated’ and suffer the toxic side effects of chemotherapy [2].

Over the past few years, using data obtained by transcriptome profiling of human breast carcinomas, it has been shown that primary breast tumors that developed metastases could be distinguished from those that remained localized. Thus, the metastatic capacity of ‘poor-prognosis’ breast tumors might be acquired by mutations at much earlier stages of tumorgenesis than was previously assumed [3].

Despite these advances in tumor classification and other classification methods which are able to discriminate between clinical relevant endpoints [4], new prognosis biomarkers are urgently needed to identify patients who are at the highest risk for developing metastases. These markers may enable oncologists to better tailor treatment strategies to individual patients [2].

In the presented study we applied Kaplan-Meier survival analysis to five datasets [5-7] comprising whole genome gene expression of 1363 patients, together with provided clinical data (Vital Status), to determine a gene's survival stratification potential. Of the roughly hundreds genes analyzed, SENP5 was the only gene that significantly stratified patients into two survival groups across all five tested datasets. Expression levels of SENP5 negatively correlate with survival of breast cancer patients.

SENP5 is a member of the SUMO-specific proteases (SENPs) family, which comprises seven partners in humans, SENP1-3 and SENP5-8 [8]. SENPs participate in SUMOylation regulation by generating mature small ubiquitin-related modifiers (SUMO) for protein conjugation (endopeptidase cleavage) and deconjugation of the targets (isopeptidase cleavage) [9]. The list of proteins subjected to SUMOylation is rapidly growing, and includes proteins localized in most subcellular compartments that are involved in the regulation of cell cycle, transcription, cell survival and death, DNA damage response, heat shock, and stress response, as well as endoplasmic reticulum and plasma membrane-associated proteins, receptors, and viral proteins [10]. The post-translational modification of SUMO to cellular substrates is vital for normal cell physiology. De-regulation of either SUMO conjugation or de-conjugation can promote cancer progression [11]. As many different signaling cascades are involved in carcinogenesis, SUMO is also tightly linked to substrates involved in cancer development and progression. In this regard SUMO is an attractive upcoming target and investigation of the manipulation of SUMO processes as a potential therapeutic intervention is gaining interest [8].

Following our findings of SENP5 to be a unique prognosis biomarker, we analyzed its role as a factor in cancer phenotypes. Here, we show that SENP5 silencing leads to inhibition of anchorage-independence growth, proliferation, migration and invasion in breast cancer cell lines.

Recently, it has been shown that the type I transforming growth factor-β (TGFβRI), which has a major role in TGFβ signaling, is SUMOylated [12]. The TGFβ superfamily plays a dual role in cancer development. In early stages of breast cancer it displays a tumor-suppressive rule; yet, in later stages, TGFβ has direct pro-tumorigenic effects through the stimulation of invasion, migration and activation of the tumor stroma [13].

We therefore followed the possibility that regulation of TGFβRI SUMOylation status accounts for changes in cancerous phenotypes which are SENP5 mediated. Our results indicate that upon SENP5 silencing, TGFβRI expression levels decrease. Further, matrix metalloprotease-9 (MMP9), a member of the MMPs which are a key determinant of loss of tissue organization in malignant transformation [14], is also repressed following SENP5 silencing.

Our findings highlight a novel, SENP5-dependant mechanism, governing metastasis and prognosis in breast cancer through TGFβ signaling, under a well regulated SUMOylation control.

## RESULTS

### Expression of SENP5 is negatively correlated with survival in breast cancer

**Figure 1:**
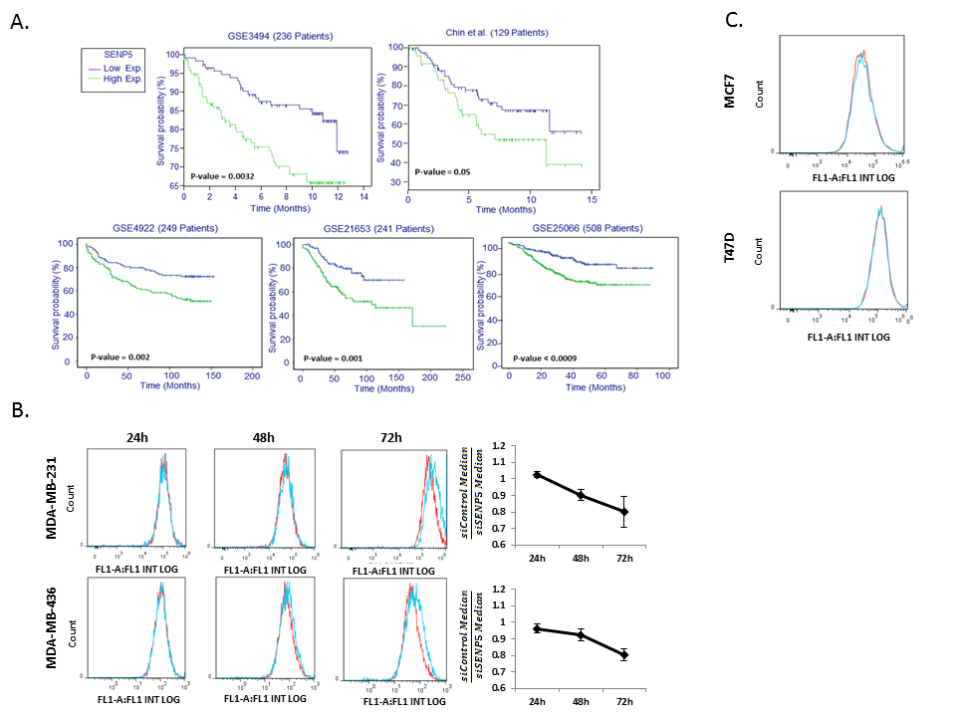
Low SENP5 levels correlates with high viability in breast cancer A. SENP5 stratifies breast cancer patients into prognosis subclasses, in five different datasets. B. Reduced proliferation following SENP5 silencing. MDA-MB-231 and MDA-MB-436 cells were treated with siControl (red line) or siSENP5 (blue line). The cells were stained with the fluorescent dye CFSE before transfection and FACS analysis was performed 24h- 72h following transfection. FACS analysis demonstrates proliferation inhibition upon SENP5 silencing in each cell line respectively. C. siSENP5 doesn't affect proliferation rate in MCF7 and T47D cells. Red line – cells treated with siControl, blue line – cells treated with siSENP5, 72h after transfection

Kaplan-Meier survival analysis was performed to stratify patients according to transcriptome quantification from five independent datasets [5-7]. We iterated across hundreds genes in the human genome, using clinical data (Vital Status), to determine a gene's survival stratification power (Figure 1A). This screen resulted in the identification of SENP5 as most significantly stratifying patients into two survival groups across all five tested datasets. High levels of SENP5 affiliate with poor prognosis while low levels of the gene affiliate with better prognosis.

These results led us to hypothesize that SENP5 has an important role in regulation of breast cancer survival. To elucidate associated mechanisms, we tested the phenotypic effect of SENP5 silencing on breast cancer cell lines.

Since breast cancer is highly dependent on estrogen receptor signaling, we next performed a multivariate analysis. Results demonstrated that ER profile couldn't account for SENP5 role as a prognostic marker (Figure S1A-E).

### Silencing of SENP5 reduces proliferation of breast cancer cells

To test the phenotypic effects of SENP5 on breast cancer cells, we first examined the proliferation rate of cancer cells transfected with control or SENP5 siRNA (siControl and siSENP5, respectively). CFSE staining of siSENP5 or siControl transfected MDA-MB-231 and MDA-MB-436 cell lines showed that silencing of SENP5 is associated with a decrease in the proliferation rates of these cells (Figure 1B). Interestingly, in two additional lines, MCF7 and T47D, silencing of SENP5 had no effect on proliferation (Figure 1C).

### Silencing of SENP5 factors on cancerous phenotypes of breast cancer cells

**Figure 2:**
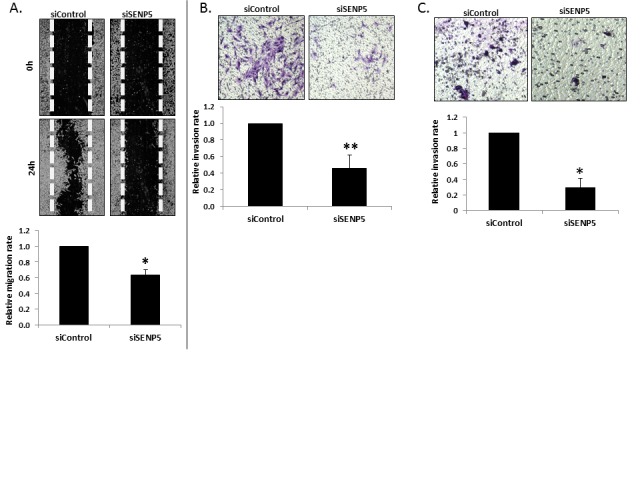
Migration and Invasion upon SENP5 silencing A. siSENP5 inhibits cell migration. MDA-MB-231 cells were treated with siControl or siSENP5 and were grown 80-90% confluence. 48h after transfection cells were scraped with a sterile micropipette tip to create a denuded zone (gap) of constant width. Wound gaps were monitored by Olympus CellSense at 0 and 24h after performing the scratch. Relative cellular migration area measured 24 hours post scratching, calculated using ImageJ software (NIH) for both siSENP5 and siControl. *P <0.01 B. Decreased invasiveness following SENP5 silencing. MDA-MB-231 cells were transfected with siControl or siSENP5. 48 hours post incubation in “Cell-Invasion-Assay-Kit” (ECM550, CHEMICON), invasion rate was measured and monitored by NIKON-TE2000. Pictures were analyzed for their relative invasion rate through the ECM matrix, using ImageJ software (NIH). **P <0.05 C. MDA-MB-436 cells were transfected with siControl or siSENP5. 48 hours post incubation in “Cell-Invasion-Assay-Kit” (ECM550, CHEMICON), invasion rate was measured and monitored by NIKON-TE2000. Pictures were analyzed for their relative invasion rate through the ECM matrix, using ImageJ software (NIH). *P <0.05

**Figure 3:**
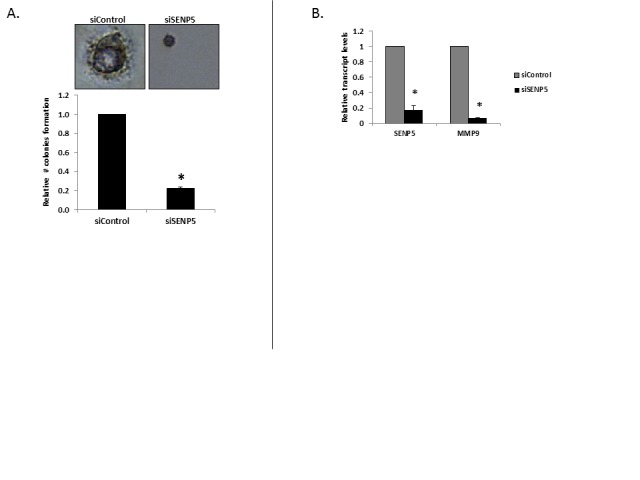
Phenotypic effect of SENP5 on breast cancer cells A. Decreased colonies formation rate following silencing of SENP5. The Soft Agar Assay for Colony Formation is an anchorage independent growth assay in soft agar. For this assay, MDA-MB-231 cells (pretreated with siControl/siSENP5) were cultured in soft agar medium for 15-25 days. Following this incubation period, formed colonies were analyzed morphologically using cell stain and quantified the number of colonies formed per well. *P <0.01 B. Reduction of MMP9 levels following SENP5 silencing. Relative transcript levels of SENP5 and MMP9 were measured by real-time qPCR (normalized to β-actin) after treatment with siControl or siSENP5. *P <0.01

In contrast to the strong effect seen in the association between SENP5 expression levels and patients' outcome, the observed reduced proliferation was mild. This led us to postulate that other factors of breast cancer aggressiveness may be associated with SENP5 activity. An important step in cancer progression is the cells' ability to degrade the basement matrix surrounding blood vessels as an early step of metastasis into distal sites. Above, we report a difference in responsiveness and non-responsiveness in the four lines we analyzed. This responsiveness matches the reported invasiveness of the tested cells. That is, MDA-MB-231 and MDA-MB-436 are invasive and metastatic lines, while MCF7 and T47D are non-metastatic. We thus employed assays of migration (wound healing assay) and invasion through reconstituted basement matrix to determine if these phenotypes are associated with SENP5 activity. Following silencing of SENP5 in MDA-MB-231 cells, both the number of cells grown into a gap (Figure 2A), and cells invading through matrix (Figure 2B) were reduced by 35% and 55% respectively. MDA-MB-436 exhibited a reduction of 70% in invasion upon silencing SENP5 (Fig 2C).Anchorage independent growth assay in soft agar is considered the most stringent assay for detecting malignant transformation of cells. We thus transfected MDA-MB-231 cells with siControl and siSENP5 and cultured them in soft agar medium for 15-25 days. Post incubation formed colonies were stained, morphologically analyzed and quantified per well, as indicated by the graph. siSENP5 treatment reduced both number and size of cells suggesting a remission in the cancerous phenotype upon SENP5 silencing (Figure 3A).

Recent studies demonstrated a key role for MMP9 in degrading the extracellular matrix, in particular the basal lamina [15]. Reduction in invasion of MDA-MB-231 cells was previously reported, in the presence of GM6001, a general inhibitor of MMPs[16]. In the present study we analyzed *MMP9* transcript levels upon SENP5 silencing. MDA-MB-231 cells were transfected with siControl or siSENP5. 48h post transfection total RNA was isolated to serve as a template for generating cDNA. qRT-PCR shows significant decrease in SENP5 transcript levels post transfection with siSENP5, as compared to siControl. Upon silencing SENP5 we saw a dramatic decrease in *MMP9* levels (Figure 3B). These results could account for the cancerous phenotype relief observed upon SENP5 silencing.

### Regulation of the cancerous phenotype by altering the SUMOylation status of TGFβRI is SENP5-mediated

**Figure 4:**
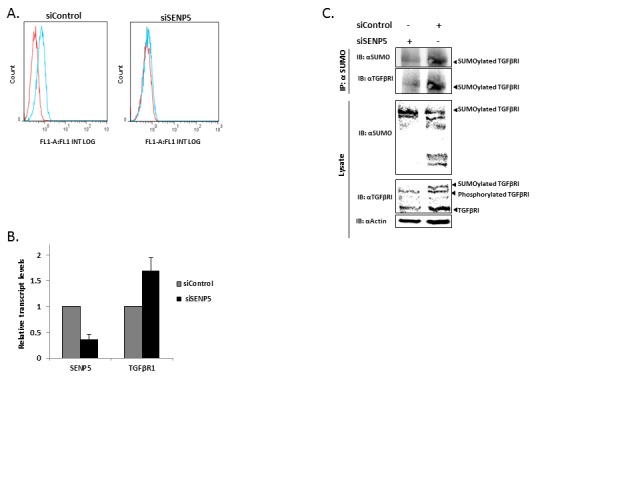
SENP5 tumorigenic effect in breast cancer induces TGFβRI post translation modification A. Silencing SENP5 leads to depletion of TGFβRI. MDA-MB-231 cells were treated with siControl or siSENP5. 48 hours after transfection, immunofluorescence staining was performed with primary TGFβRI antibody and secondary FITC antibody, and then analyzed by flow cytometer (FACS). Red line – cells were treated only with the secondary antibody as a negative control. Blue line – cells were treated with both antibodies. B. Depletion of TGFβRI following SENP5 silencing is not due to a decrease in TGFβRI transcript levels. Relative transcript levels of SENP5 and TGFβRI were measured by real-time qPCR (normalized to β-actin) after treatment with siControl or siSENP5. C. SENP5 silencing results in TGFβRI protein levels decrease as well as loss of TGFβRI SUMOylated form. Lysates of MDA-MB-231 cells, treated with siControl or siSENP5, were immunoprecipitated with anti-SUMO(1/2/3) and immunoblotted with antibody against TGFβRI. In addition, endogenous expression levels of TGFβRI and SUMO were measured for the pre-treated lysates.

The regulatory cytokine TGFβ exerts tumor-suppressive effects that cancer cells must elude for malignant evolution. Yet, paradoxically, TGFβ also modulates processes such as cell invasion, immune regulation, and microenvironment modifications that cancer cells may exploit to their advantage [17]. It has recently been shown that SUMO proteins, which primarily modify nuclear proteins and regulate their function, are conjugated to TGFβRI in a regulated manner. TGFβRI SUMOylation modulates its function [12]. Since SENP5 modulates deSUMOylation and as we show, correlates with cancerous phenotype control, we aimed to identify a possible SENP5-TGFβRI interplay. MDA-MB-231 cells were transfected with siControl or siSENP5. 48h post transfection immunofluorescence staining was performed with the primary antibody TGFβRI and secondary anti-Rabbit-FITC antibody, and then analyzed using a flow cytometer (FACS) demonstrating a reduction in TGFβRI protein levels due to siSENP5 treatment (Figure 4A). Next, we showed that the reduction in TGFβRI protein levels demonstrated following SENP5 silencing, in not due to transcript depletion (Figure 4B). We therefore examined whether post translational modifications, in particular SUMOylation, could account for observed phenotypic alternations, which are TGFβRI mediated. To characterize the mechanism by which SENP5 regulates the phenotype through TGFβRI SUMOylation regulation, we used co-immunoprecipitation assay.

MDA-MB-231 cells were transfected with siControl or siSENP5. 48h post transfection, in SENP5 silenced cells, we identified reduction in the SUMOylated form of TGFβRI in the immunocoplexes. The input lysates blotted to anti-SUMO, revealed different patterns of SUMOylated protein upon SENP5 silencing, while exposure to anti-TGFβRI confirmed a reduction in the total pool of TGFβRI, the SUMOylated-TGFβRI and a TGFβRI form which could comprise the phosphorylated form of TGFβRI. Anti-Actin served as a loading control (Figure 4C).

## DISCUSSION

As biomedical research bands together to provide more quality data from multi patient, high-throughput experimentation, much of these data are already mature enough to re-shape bench to bedside advancement.

Breast cancer is arguably the most genomically studied disease. Using different breast cancer datasets, we studied gene–expression profiles of 1363 breast cancer patients and screened these data for appropriate candidate genes to serve as biomarkers to stratify clinical groups of patients based on their expression profiling. Using this approach, we identified SUMO-specific protease 5 (SENP5), as highly significant in stratifying patients into outcome groups across tested datasets. Results demonstrate that low expression of SENP5 correlates with high survival index. We next demonstrated that ER profile couldn't account for SENP5 role as a prognostic marker (Figure S1A-E).

Such novel prognosis biomarkers are urgently needed to identify patients who are at the highest risk for developing metastases, thus enabling oncologists a personalization of treatment strategies according to molecular features of tested patients [2].

Post-translational modifications of proteins by the small Ubiquitin-like modifier SUMO is a central mechanism, regulating numerous biological processes, including trafficking, transcription, DNA repair and replication, as well as mitotic and meiotic chromosome behavior [18-20]. SUMOylation is a highly dynamic process which is tightly regulated by a fine balance between conjugating and de-conjugating enzyme activities. Covalent modification of proteins by SUMO is reversible. SUMO is covalently attached to lysine residues in substrate proteins in a process similar to Ubiquitination[21]. SUMO conjugation requires an E1-activating enzyme (Aos1/Uba2) and an E2-conjugating enzyme (Ubc9), and SUMOylation of specific substrates may be stimulated by the action of diverse E3 ligases [22-24].

SENP proteins share dual roles in the SUMOylation pathway. First, they are responsible for the initial processing of SUMO precursors to generate a C-terminal diglycine motif required for conjugation. Second, these proteases execute the deconjugation reaction that removes SUMO from high-molecular-weight SUMO conjugates.

Loss of balance between SUMOylation and deSUMOylation has been reported in a number of studies in a variety of disease types including cancer [8].

Our findings demonstrated a pivotal role of SENP5 in determining prognosis in breast cancer patients, according to their SENP5 expression profiles. We thus investigated SENP5 role in altering cancerous phenotypes, employing RNA interference approach.

We used CFSE staining at different time points (24, 48 and 72h) and quantified proliferation rates of MDA-MB-231 and MDA-MB-436. We showed that upon SENP5 silencing, proliferation rates were only slightly reduced, with an intriguing lack of impact on proliferation rates in MCF7 and T47D, which are ER^+^ and are weakly invasive breast cancer cell lines. These data led us to ascertain that proliferation rates could not account for the differences observed in clinical samples. Consequently, we investigated whether silencing of SENP5 may interfere with cell migration or cell invasion. As MCF7 and T47D are non-invasive cell lines, we assessed these tumorigenic phenotypes in the MDA-MB-231 ER^−^ metastatic cell line, as well as in MDA-MB-236.

A wound healing assay demonstrated a 35% reduction in MDA-MB-231 migration mediated by siSENP5. Invasion of MDA-MB-231 transfected with siSENP5 reduced in 55%, where MDA-MB-236 exhibits 70% reduction (Fig 2C). A soft agar assay further assessed SENP5 involvement in tumor phenotype alterations. This assay showed reduction both in numbers and in sizes of colony formation upon SENP5 silencing.

These experiments, which demonstrated that tumorigenesis may be impaired with the introduction of siSENP5, combine with previously shown findings demonstrating that SENP5 may regulates the SUMOylation status of yet unknown substrates [10]. This combination led us to look for possible candidates that might be a direct or an indirect SENP5 target and that may be involved with demonstrated behavior.

It has been recently shown that the type I transforming growth factor-β (TGFβRI), which has a major role in TGFβ signaling, is SUMOylated [12]. The TGFβ superfamily is considered both a tumor suppressor (initial stages) as well as a stimulating factor (later stages) in breast cancer [25]. Moreover, TGFβ is frequently overexpressed in breast cancer and its expression correlates with poor prognosis and metastasis [26-29].

Kang and colleague previously suggested that TGFβRI SUMOylation enhances invasion and metastasis of Ras-transformed cells. In this model, TGFβRI SUMOylation contributes to tumor progression by enhancing tumor cell extravasation, survival and/or growth at the metastatic site. Recent data demonstrated that blocking TGFβ signaling with a dominant-negative form of TGFβRI or Smad4 knockdown in human breast cancer cell lines decreased the ability of these cells to generate lung metastases when implanted as mammary tumors in mice [17].

These results are in agreement with our findings which demonstrated reduction in migration and invasion upon SENP5 silencing and may indicate some interplay between TGFβRI and SENP5.

TGFβ signaling plays a critical role in the regulation of cell growth, differentiation, and development in a wide range of biological systems. The mechanisms that lead to receptor activation and gene expression in response to TGFβ are generally understood [30]. Binding of TGFβ to a complex of two type I and two type II kinase receptors, i.e. TGFβRI and TGFβRII, confers TGFβRI activation and consequent direct C-terminal phosphorylation of Smad2 and Smad3 by TGFβRI. The activated Smads then associate with Smad4 and translocate into the nucleus to regulate transcription of target genes [31, 32].

Yet, less is known about the regulation of TGFβ receptors by post-translational modifications. We hypothesized that regulation of TGFβRI SUMOylation may account for the observed phenotypic changes mediated by SENP5.

To study this possible association between SENP5 and TGFβRI, we first used FACS analysis and showed depletion in TGFβRI levels upon SENP5 silencing. qRT-PCR demonstrated that this reduction in TGFβRI upon SENP5 silencing does not correlate with transcription depletion, which may indicate that these changes are the result of translational and/or post translational events.

By employing a co-immunoprecipitation assay we further strengthened this hypothesis: results indicated that siSENP5 led to reduction of total TGFβRI, whereas the SUMOylated fraction of TGFβRI has completely disappeared (western blot analysis using anti-TGFβRI and anti-SUMO, respectively). It has been shown that TGFβRI autophosphorylation plays a role in its SUMOylation: increased kinase activity together with increased phosphorylation contribute remarkably to TGFβRI SUMOylation, by targeting a Lys389 residue [12]. In agreement with these findings, we identified that upon SENP5 silencing a reduction in the phosphorylated fraction of TGFβRI is also detected (Figure 4C): Immunoblot analysis reveals depletion in a band corresponding to the expected phospho-TGFβRI, detected by specific anti-TGFβRI antibody.

We speculated that by silencing SENP5 we will force overexpression of total TGFβRI as well as induction of the SUMOylated form of TGFβRI. As this was not the case, we speculated that SENP5- TGFβRI share other interactions rather than a SENP / Target model. One possible assumption is that there is another partner which regulates TGFβRI stability in a direct or indirect fashion, upon its intrinsic SENP5 mediated deSUMOylation. In such a scenario, SUMOylation of the unknown component, upon SENP5 silencing, activates it to promote TGFβRI degradation, while SENP5 deSUMOylation abolishes its TGFβRI regulation control and in turn stabilizes TGFβRI levels.

Support to the assumption that SENP5-TGFβRI interaction is mediated by an additional partner calls for further investigation. However, this assumption is supported by the fact that TGFβRI and SENP5 were previously reported to interact with different SUMO partners: TGFβRI is known to be regulated by SUMO1 whereas SENP5 was previously reported to be associated with SUMO2/3 [12].

Alternatively, as SENP5 displays both C-terminal hydrolase and isopeptidase activities, SENP5 may mediate the cleavage activity of amide bond between the C-terminus of the mature SUMO and the ε-amino group of the target lysine within the TGFβRI substrate. During SUMO metabolism, Ulp/SENPs catalyze three distinct processes: processing, de-conjugation, and chain editing [33]. Like Ubiquitin, SUMO proteins are expressed as precursor proteins that carry a C-terminal extension of variable length (2-11 amino acids) found after a conserved di-glycine motif. To function as a modifier of target proteins, the C-terminal di-glycine motif of the SUMO proteins must be exposed by the action of SUMO specific protease [34]. SENP5 has demonstrated SUMO3 C-terminal hydrolase activity, but does not process SUMO1 *in vitro*. In addition, SENP5 demonstrated isopeptidase activity with SUMO2 and SUMO3 conjugates but not against SUMO1 conjugates *in vivo* [35]. If that is the case, by silencing SENP5, the cleavage on TGFβRI C-terminus is blocked, thus inhibiting its SUMOylation process.

A third possibility is that while TGFβRI is being modified by poly-SUMO residues which lead to Ubiquitination and consequently to degradation, SENP5 disrupts the poly-SUMO chain conversion and abrogates Ubiquitination which in turn stabilized TGFβRI. Although TGFβRI has demonstrated SUMO1 regulation (Lys389 residue) which does not act as a link in elongating chains, TGFβRI SUMOylation could alternatively be mediated through other acceptor Lysine permitting SUMO2/3 processing [36]. SUMO2/3 can form conjugated chains through a single conserved, due to a consensus SUMO acceptor site which is somewhat analogous to Ubiquitin and may form elongating chains [37]. Since SUMOylation can confer different fates, the poly-SUMO can serve as Ubiquitin target, thus steering the SUMOylated protein to proteasome degradation [36]. Under those circumstances, silencing SENP5 results in TGFβRI proteasome-mediated degradation, which is in agreement with our results.

Recent studies revealed a key role for MMP9 in TGFβ-induced invasion. MMP9 is able to degrade the extracellular matrix, in particular the basal lamina [15]. Various matrix components that can be degraded by MMP9 are in fact produced by the epithelial cells themselves. However, besides degradation of extracellular matrix MMPs can also activate growth factors, and cleave adhesion molecules, such as E-cadherin [38]. It is likely that these functions of MMP9 also contribute to TGFβ-induced invasion [25].

Moreover, reduction in invasion of Matrigel by tumor cells was previously reported, in the presence of GM6001, a general inhibitor of matrix metalloproteinases (MMPs), suggesting an important role of MMPs in invasiveness of MDA-MB-231 cells [39].

Although it is well documented that MMP9 is required for tumor progression [40, 41], the source of MMP9 is still a subject of discussion. It has been shown that MMP9 is secreted by tumor cells [42-44] as well as by cells residing in stromal compartments [45, 46].

Analysis of TGFβ-induced mRNA expression identified *MMP1*, *MMP2*, and *MMP9* among the strongest TGFβ-responsive genes [25].

However, MMPs may also have anti-tumor actions. The function appears in particular as an issue in targeting of MMP9 [47]. Tumors of K14-HPV16 mice in a null MMP9 background were found to be more aggressive, indicating that MMP9 inhibits certain aspects of tumor progression [48]. On the other hand, knockdown of MMP9 exhibited depletion in metastasis of TGFβRI-expressing cells [39].

Taken together, it appears that basal invasion depends on other factors induced by TGFβ, whereas MMPs are necessary for overt invasion [25].

**Figure 5:**
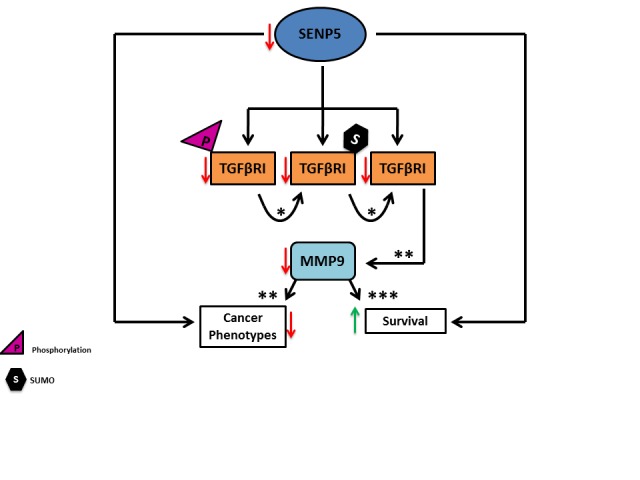
A decrease in SENP5 levels mediates TGFβRI post translation modifications deSUMOylation follows TGFβRI de-phosphorylation, which results in MMP9 depletion. This, in turn, contributes to an attenuate in aggressiveness of cancerous phenotypes. *[12] ** [25] *** [48]

In accordance with this paradigm, we demonstrated that SENP5 silencing induces a dramatic reduction in *MMP9* transcript levels probably by depletion of TGFβRI. These results suggest a novel mechanism by which SENP5 modulates TGFβRI to transduce the oncogenic signal, by attenuates its MMP9 transcript levels (Figure 5).

Our findings highlight a novel, SENP5-dependant mechanism, clinical research based, governing metastasis and prognosis in breast cancer through TGFβ signaling, under a well regulated SUMOylation control.

## MATERIALS AND METHODS

### Kaplan-Meier survival analysis

To determine a gene's survival stratification power, Genes were tested across five datasets [5-7]: GSE3494, GSE3922, GSE21653 and GSE25066 Gene expression levels were clustered using K-means clustering to stratify the patients into two groups. Genes that had significant p-values (<0.05) were then chosen as good biomarkers for prognosis. The results were then compared in all five datasets to identify overlapping genes.

### Cell lines

MDA-MB-231, MDA-MB-436, T47D and MCF7 were purchased from ATCC. Cells were grown at 37°C with 5% CO2 in RPMI 1640 (T47D) or DMEM (MCF7, MDA-MB-231, MDA-MB-436) medium supplement with 2 mM L-glutamine, 1.5 g/L sodium bicarbonate, 4.5 g/L glucose, 10 mM HEPES, 1.0 mM sodium pyruvate and 10% fetal bovine serum (FBS). T47D cells were also supplement with 0.2U/ml bovine insulin.

### siRNA mediated SENP5 silencing

siRNA duplexe targeted to SENP5 gene purchased from Invitrogen (Life technologies Co.GI,NY,USA).sense:5'GAACAUCGUUCUAAUACCAUGUUCA3'antisense: 5'UGAACAUGGUAUUAGAACGAUGUUC3'. Cells were transfected using JETprime reagent (PolyPlus-transfection™, NY, NY, USA) according to the manufacturer protocol. Control transfection for the specificity of the SENP5 silencing effect was done using Invitrogen universal negative control, medium GC content. All transfections were done in final siRNA concentration of 1nM.

### RNA extraction and cDNA synthesis

Total RNA was isolated from the cultured cells following transfection using Tri reagent (Ambion® Life technologies Co. GI, NY, USA) according to manufacturer's instructions.Up to 1 μg of RNA was used for cDNA synthesis using High Capacity cDNA Reverse Transcription kit (Applied Biosystems, Life technologies Co.GI, NY, USA) according to the manufacturer protocol.

### Real Time qPCR

Real Time qPCR reactions are performed to quantitate SENP5 gene expression. Each PCR reaction contains 2 μl of serially diluted cDNA samples, 10 pmoles of each forward and reverse primer, complementary to the tested gene – SENP5, MMP9, TGFβRI or β-actin, as a loading control, and 10 μl KAPA Syber FAST ABI Prism qPCR Kit (KapaBiosystems Inc. Woburn, MA, USA). Reactions were run on 7900HT Real Time PCR (Applied Biosystems, Life technologies Co.GI, NY, USA) instrument in FAST mode with standard curve program keeping the manufacturer defaults. The primers sequences are as follows: SENP5:F:5'TGCTAGATCACCTCGTCTTCA3'R:5'AGTGCTTAGTGGTTTTCATGATA3'. MMP9:F:5'ACGCAGACATCGTCATCCAGT3'R: 5'GGACC ACAACTCGTCATCGTC3'.TGFβRI:F:5'AAGGTACATGGCCCCTGAAGTT3'R:5'CGTCGAGCAATTTCCCAGAA3'.

βactin:F:5'AGCGAGCATCCCCCAAAGTT3'R:5'GGGCACGAAGGCTCATCATT3'

### Cells proliferation assay

Proliferation was tested by CFSE (5,6-carboxylfluorescein diacetatesuccinimidyl ester), as follows: 10^7^ cells/ml were incubated in a suitable medium containing 5μM CFSE for 20 min, followed by quenching with 50% fetal bovine serum. The cells were then washed twice and 2x10^5^ cells cultured in 3.5cm plates, and transfected with control or SENP5 siRNA as detailed above. FACS (Fluorescence Activated Cell Sorter) analysis (Gallios) was performed 24h-72h following transfection. As the fluorescence intensity of CFSE-stained cells halves with each cell division, cells that have divided are easily identified and enumerated by flow cytometry. The median of each experimental group was measured by FlowJo software (Tree-Star, Ashland, USA). A higher value of the median means that the CFSE color less faded and therefore the examined population less divided. In order to normalize the different results we divided the median of the population treated with siControl by the one treated with siSENP5.

### Invasion assay

Invasiveness was examined with a Cell-Invasion-Assay-Kit (ECM550, CHEMICON - Millipore Co. Billerica, MA, USA) which has two chambers. In the top chamber we seeded cells in a serum-free medium, while at the bottom there was a complete medium. The two chambers are separated by an ECMatrix™ coated membrane that simulates the basement membrane on the blood vessels. The ECM layer occludes the membrane pores (8μm pore size), blocking non-invasive cells from migrating through. Invasive cells, on the other hand, migrate through the ECM layer and cling to the bottom of the polycarbonate membrane. 24h after transfection, 3x10^5^ cells were suspended in 300μl of serum free media and plated on the membrane inserts of an Invasion Chamber. The cells were incubated for 48h, after which the cells that did not invade through the pores were removed with a cotton swab. Cells on the lower surface of the membrane were stained for visualization.

### Wound healing assay

Wound healing assay is a straight forward method to study cell migration *in vitro*. This method is based on the observation that, upon creation of a new artificial equally gap zone on an 80-90% confluent cell monolayer. Cellular debris were washed with PBS. The cells on the edge of the newly created gap move toward the opening to close the “scratch”[49]. Cell migration into the gap was monitored and photographed during 24h. Calculating the areas was done using ImageJ software (NIH).

### Soft Agar Assay

The Soft Agar Assay for Colony Formation is an anchorage independent growth assay in soft agar, which is considered the most stringent assay for detecting malignant transformation of cells. For this assay, MDA-MB-231 cells (pretreated with siControl/siSENP5) were cultured in soft agar medium for 15-25 days. Following this incubation period, formed colonies were analyzed morphologically using cell stain and quantified the number of colonies formed per well.

### Immunoflourescence staining

Cells were harvested by trypsinization 48h post transfection (with siControl/siSENP5), washed twice with PBS and fixed with 70% ethanol overnight at 4°C. The fixed cells were rehydrated once in PBS, and washed twice with a FACS buffer (PBS containing 0.5% bovine serum albumin). Presence of TGFβRI was detected by using 1:250 (diluted in FACS buffer) rabbit polyclonal antibody against TGFβRI (Bioss, Inc. Woburn, MA, USA). The cells were stained with 25μl of anti-TGFβRI antibody for 45min at 4°C. The cells were rinsed three times with FACS buffer and were counterstained for 45min at 4°C with mouse anti-rabbit IgG (heavy and light chain)–FITC conjugated antibody (Santa Cruze-Biotec. Dallas, Texas, USA) diluted 1:15 in FACS buffer. The cells were rinsed three times with FACS buffer and were resuspended in 0.5 ml of FACS buffer for FACS analysis (Gallios).

### Immunoprecipitation

MDA-MB-231 Cells transfected with siControl/siSENP5 were lysed in cell lysis buffer (1% Triton X-100, 150 mM NaCl, 50 mM Tris-HCl, pH 7.4, 1 mM EDTA, and protein inhibitor cocktail (Sigma Aldrich Co. Rehovot, Israel) on ice, 48h after transfection. Lysates were incubated with rabbit anti-SUMO1/2/3 antibody (Boston Biochem® MA, USA) with rotation overnight at 4°C. Immunocomplexes were precipitated with protein Aagarose beads (Millipore Co. Billerica, MA, USA) for 1h, with rotation at 4°C. Beads were collected by slow centrifugation, washed 3 times with lysis buffer and analyzed by SDS-PAGE followed by detection with specific antibody.

### Western Blotting

MDA-MB-231 Cell extracts were prepared with cell lysis buffer (1% Triton X-100, 150 mM NaCl, 50 mM Tris-HCl, pH 7.4, 1 mM EDTA, and protein inhibitor cocktail (Sigma Aldrich Co. Rehovot, Israel) on ice, 48h after transfection. Following SDS polyacrylamide gel electrophoresis (SDS-PAGE) separation, proteins were transferred to nitrocellulose membranes and blocked with 5% low fat milk. Membranes were incubated with rabbit anti-TGFβRI (Bioss) or anti-SUMO1/2/3 (Boston Biochem® MA, USA) primary antibodies, washed with PBS containing 0.001% Tween-20 (PBST) and incubated with the appropriate horseradish peroxidase-conjugated secondary antibody, Goat-anti-rabbit-HRP (Jackson Immuno-Research, Sufflok, England). After washing in PBST, membranes were subjected to enhanced chemiluminescence detection analysis.

## SUPPLEMENTARY FIGURE


