# Supplementary material for: SENP5 mediates breast cancer invasion via a TGFβRI SUMOylation cascade
Source: Oncotarget. 2014 Feb 18;5(4):1071–82. doi: 10.18632/oncotarget.1783 (PMC4011584; doi:10.18632/oncotarget.1783)
Supplement: Supplementary file 1 [file oncotarget-05-1071-s001.pdf]

A.

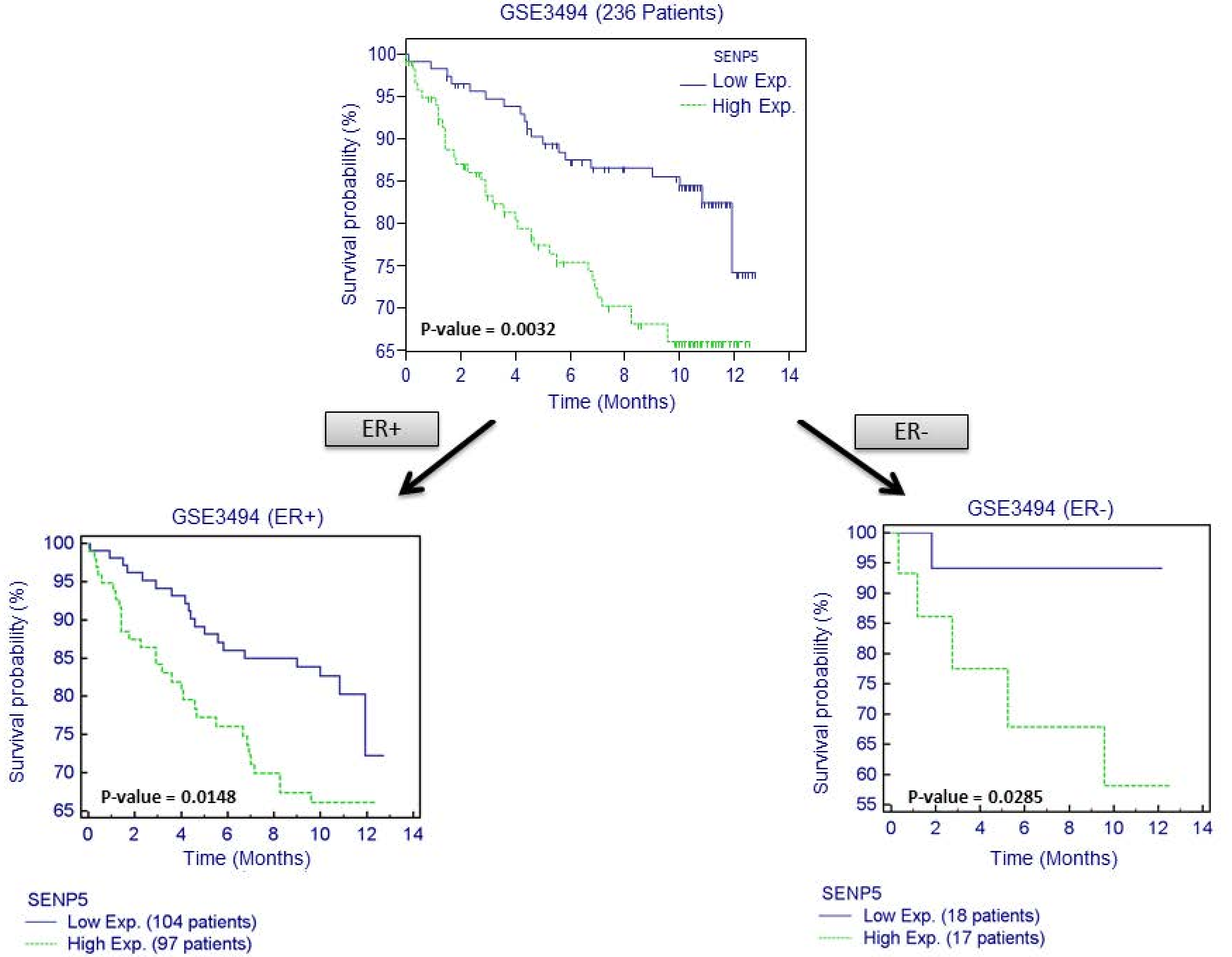
GSE3494 (236 Patients)
SENP5
Low Exp.
High Exp.
Survival probability (%)
P-value = 0.0032
Time (Months)
ER+
ER-
GSE3494 (ER+)
Survival probability (%)
P-value = 0.0148
Time (Months)
SENP5
Low Exp. (104 patients)
High Exp. (97 patients)
GSE3494 (ER-)
Survival probability (%)
P-value = 0.0285
Time (Months)
SENP5
Low Exp. (18 patients)
High Exp. (17 patients)

B.

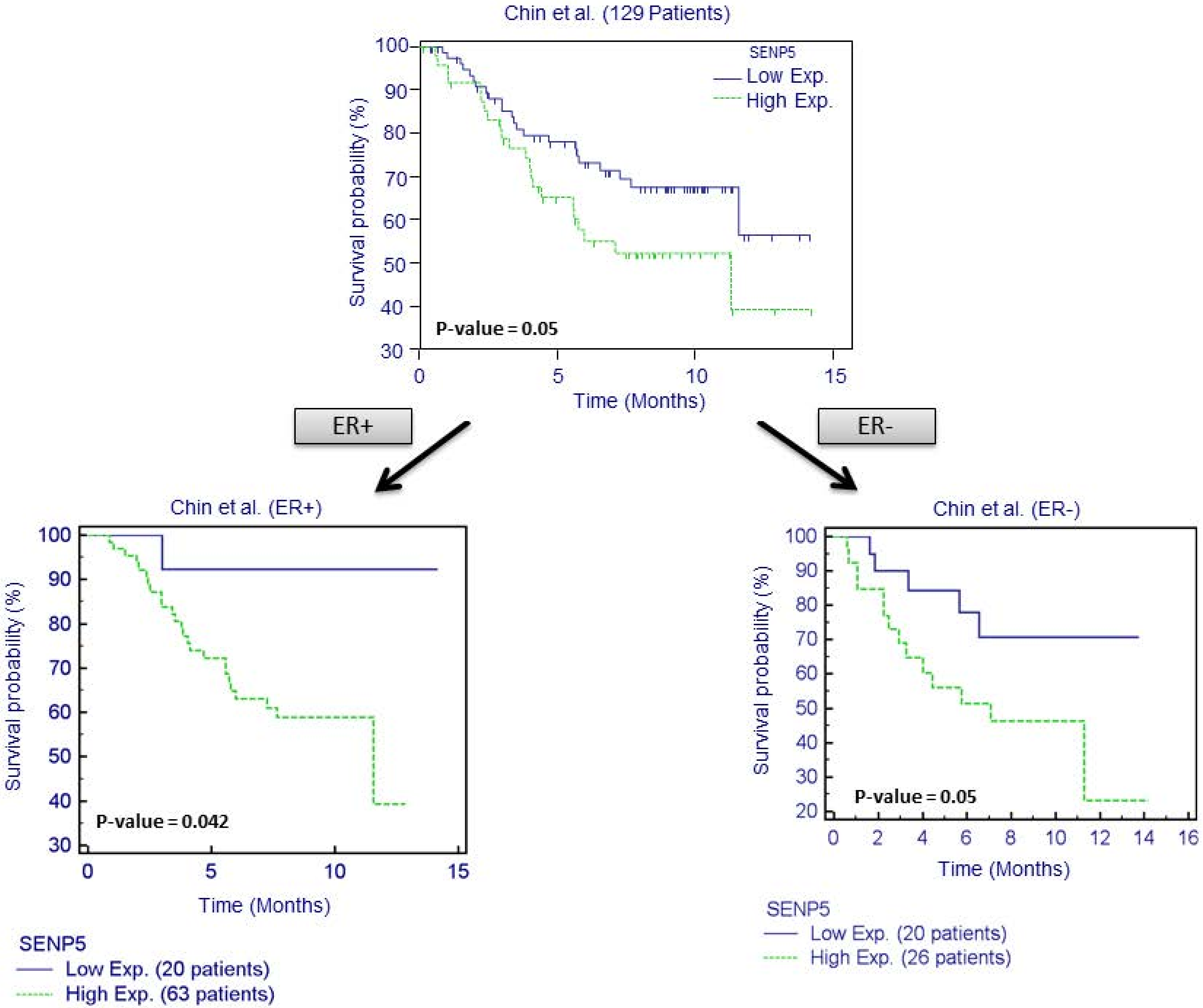
Chin et al. (129 Patients)
SENP5
Low Exp.
High Exp.
Survival probability (%)
P-value = 0.05
Time (Months)
ER+
ER-
Chin et al. (ER+)
Survival probability (%)
P-value = 0.042
Time (Months)
SENP5
Low Exp. (20 patients)
High Exp. (63 patients)
Chin et al. (ER-)
Survival probability (%)
P-value = 0.05
Time (Months)
SENP5
Low Exp. (20 patients)
High Exp. (26 patients)

C.

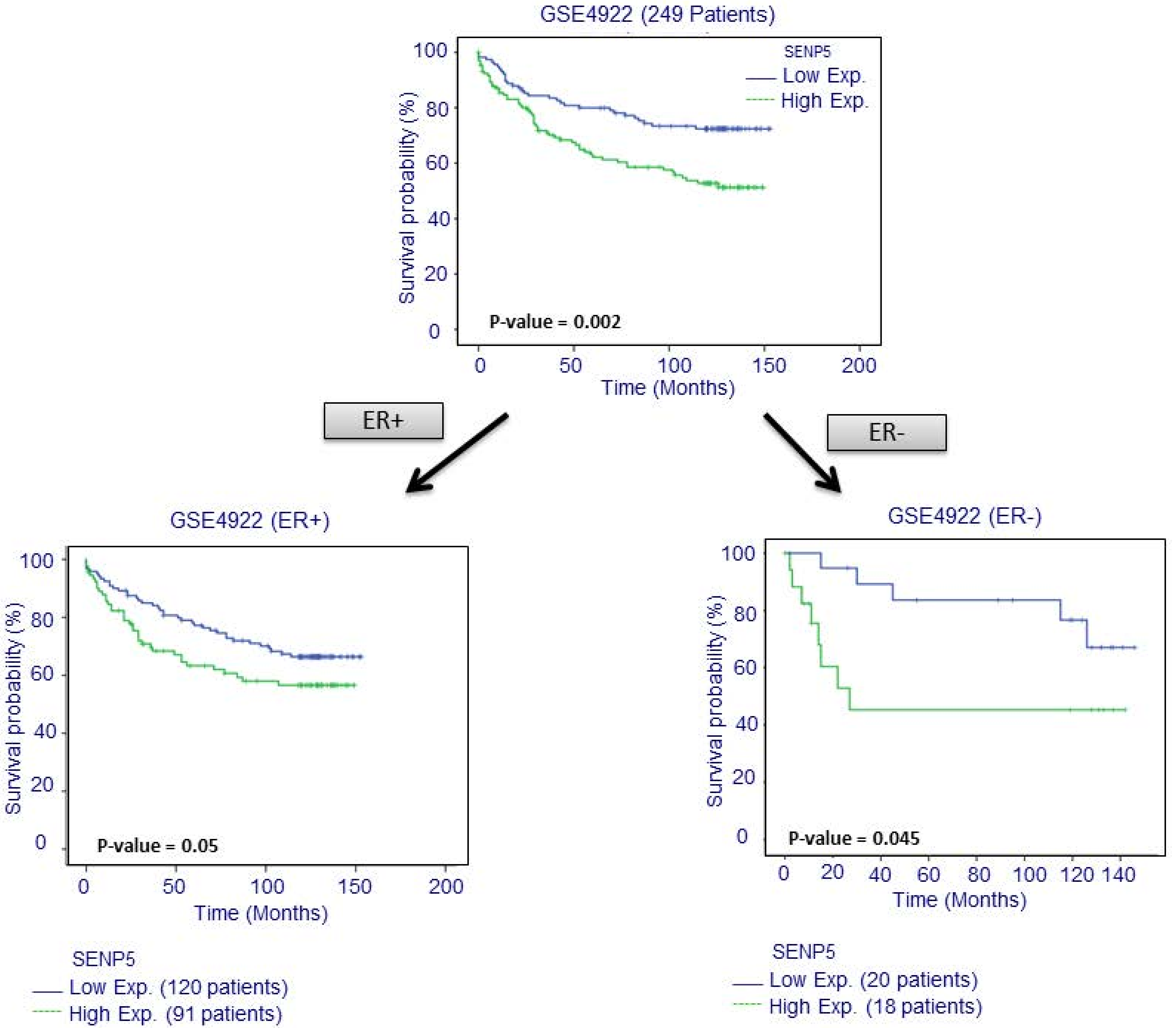
GSE4922 (249 Patients)
SENP5
Low Exp.
High Exp.
Survival probability (%)
100
80
60
40
20
0
P-value = 0.002
0
50
100
150
200
Time (Months)
ER+
ER-
GSE4922 (ER+)
Survival probability (%)
P-value = 0.05
Time (Months)
SENP5
Low Exp. (120 patients)
High Exp. (91 patients)
GSE4922 (ER-)
Survival probability (%)
P-value = 0.045
0
20
40
60
80
100
120
140
Time (Months)
SENP5
Low Exp. (20 patients)
High Exp. (18 patients)

D.

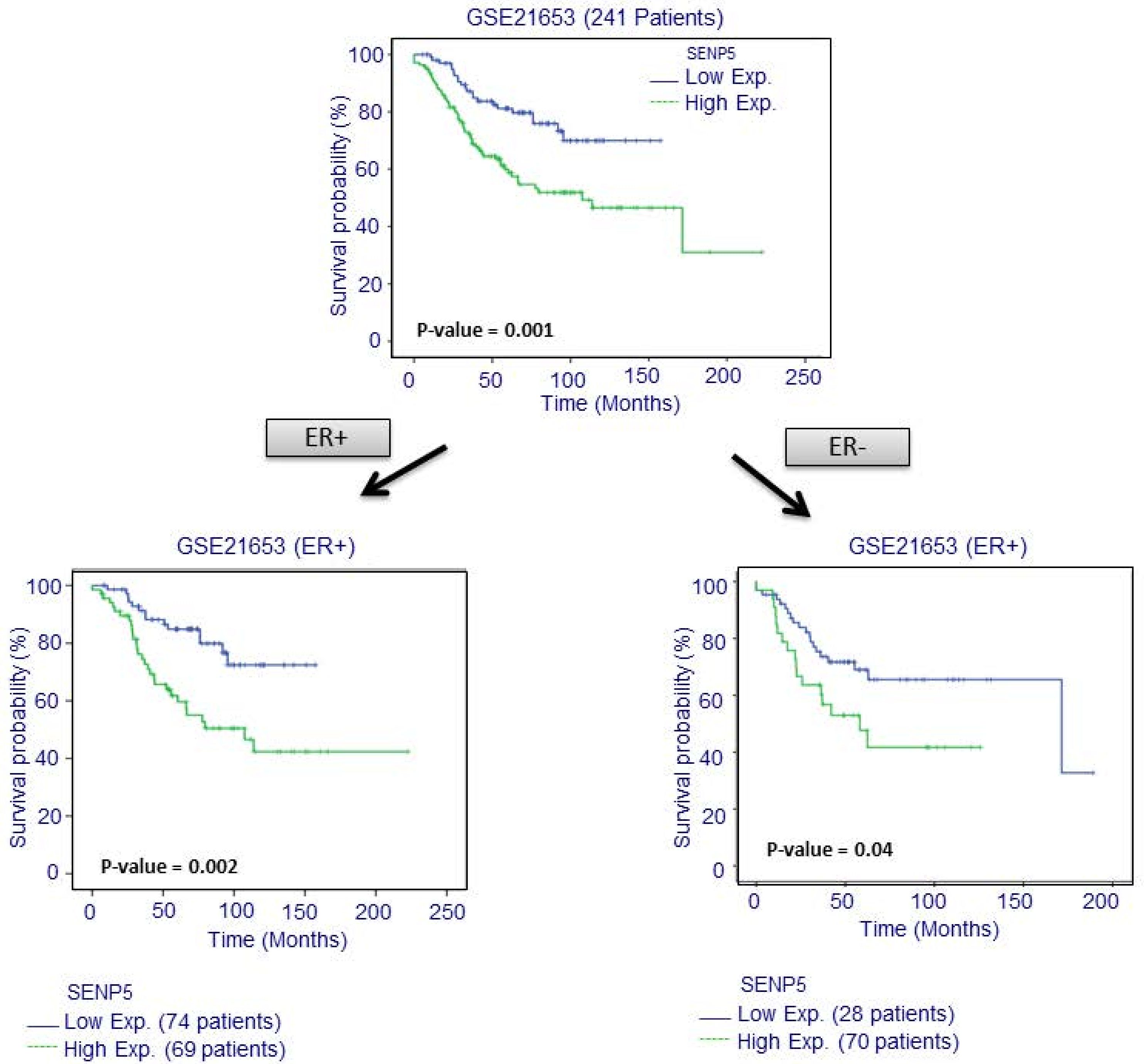
GSE21653 (241 Patients)
SENP5
Low Exp.
High Exp.
Survival probability (%)
100
80
60
40
20
0
P-value = 0.001
0
50
100
150
200
250
Time (Months)
ER+
ER-
GSE21653 (ER+)
Survival probability (%)
P-value = 0.002
Time (Months)
SENP5
Low Exp. (74 patients)
High Exp. (69 patients)
GSE21653 (ER+)
Survival probability (%)
P-value = 0.04
Time (Months)
SENP5
Low Exp. (28 patients)
High Exp. (70 patients)

E.

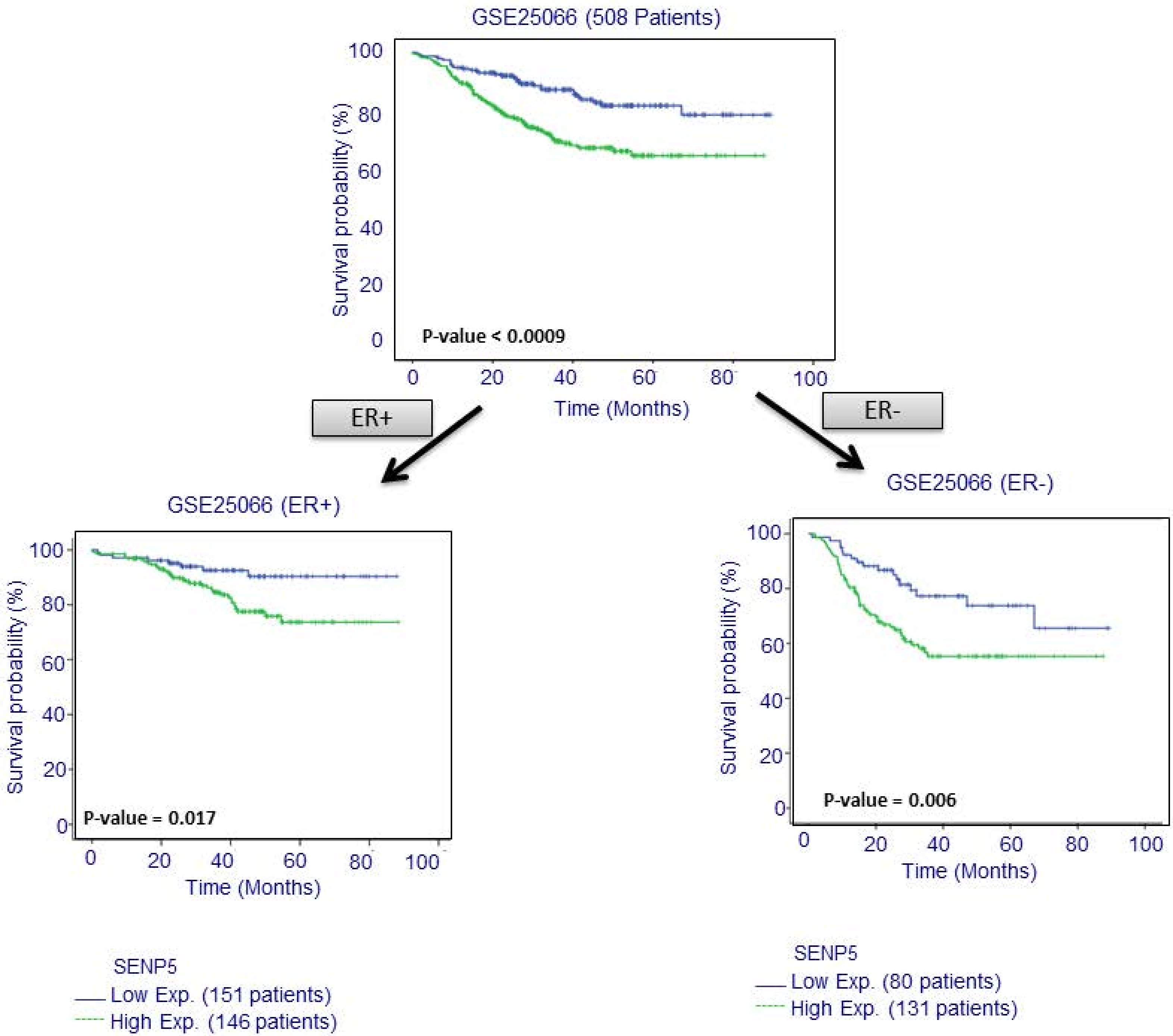

GSE25066 (508 Patients)
Survival probability (%)
Time (Months)
P-value < 0.0009
ER+
ER-
GSE25066 (ER+)
P-value = 0.017
GSE25066 (ER-)
P-value = 0.006
SENP5
Low Exp. (151 patients)
High Exp. (146 patients)
SENP5
Low Exp. (80 patients)
High Exp. (131 patients)

Figure S1. Low SENP5 levels correlates with high viability in breast cancer

A. – E. SENP5 stratifies breast cancer patients into prognosis subclasses, in five different datasets, independently with their ER context.
